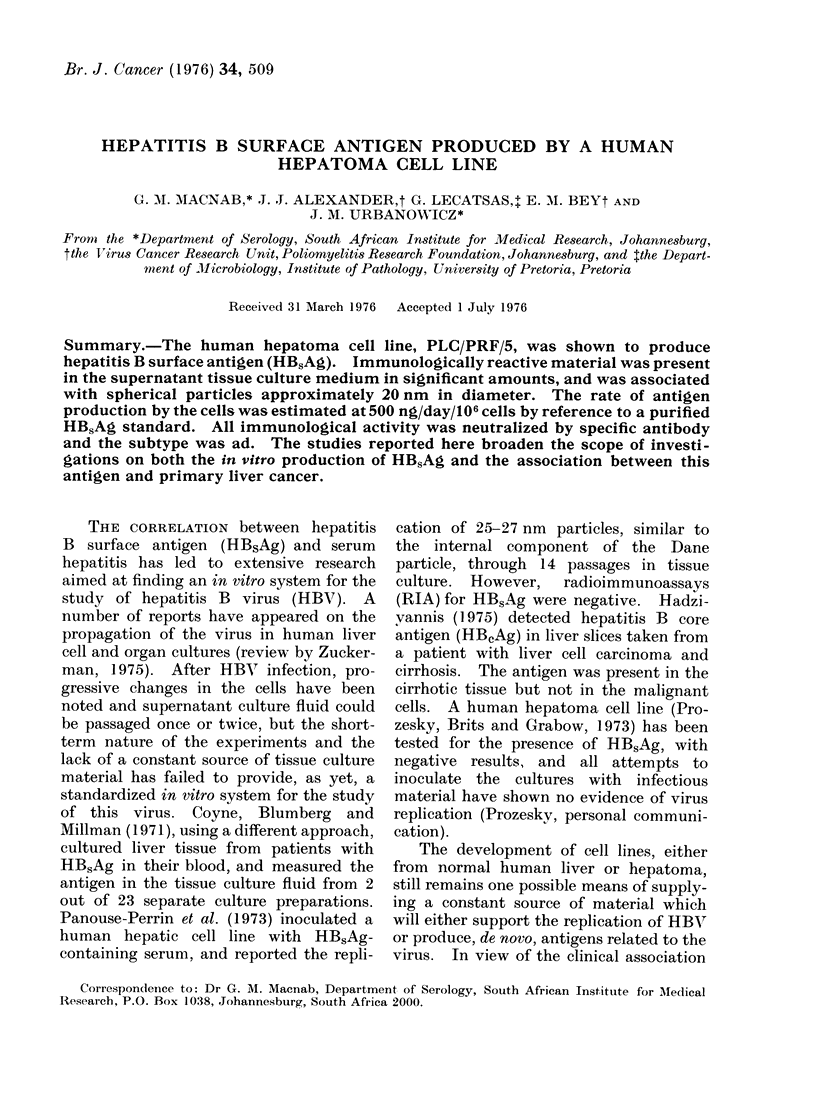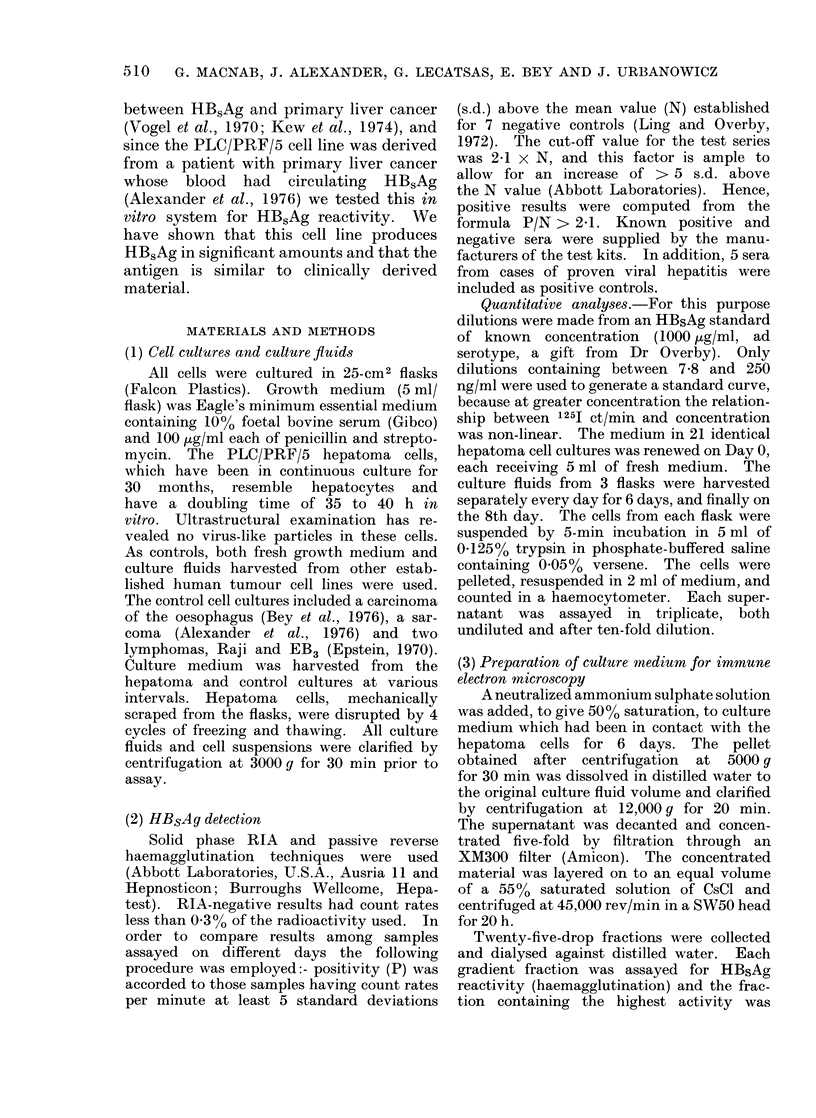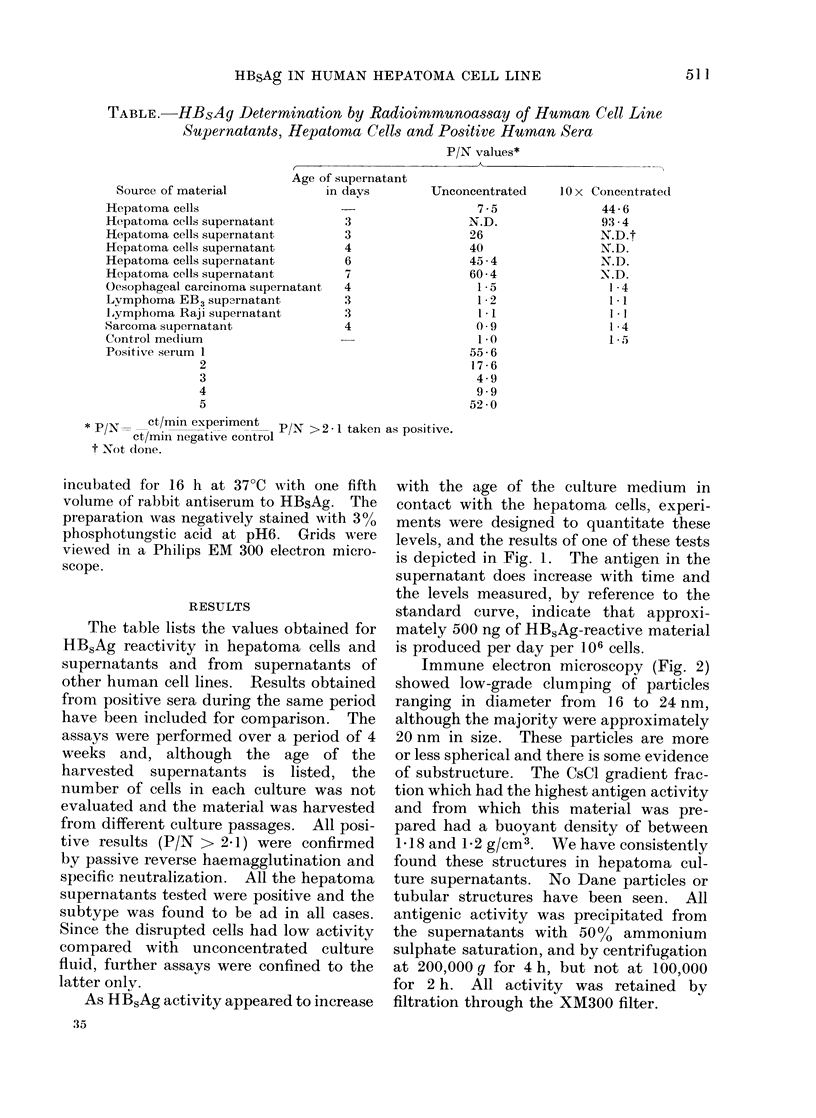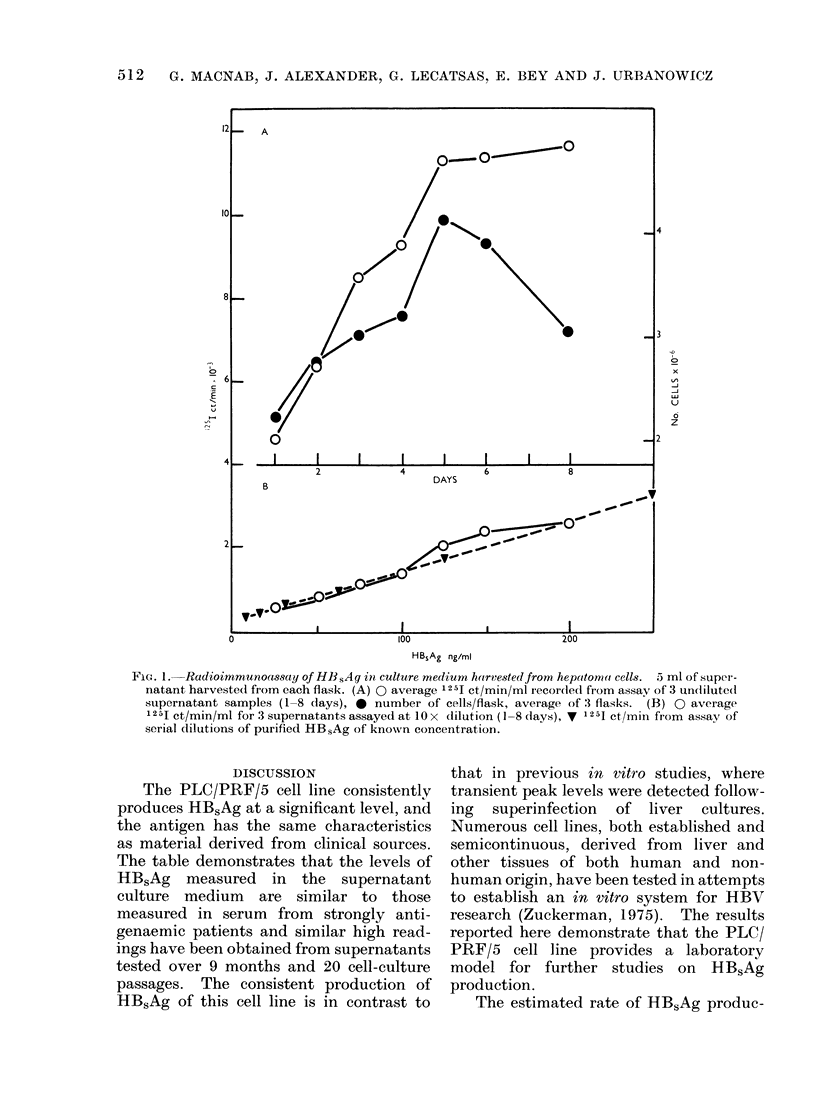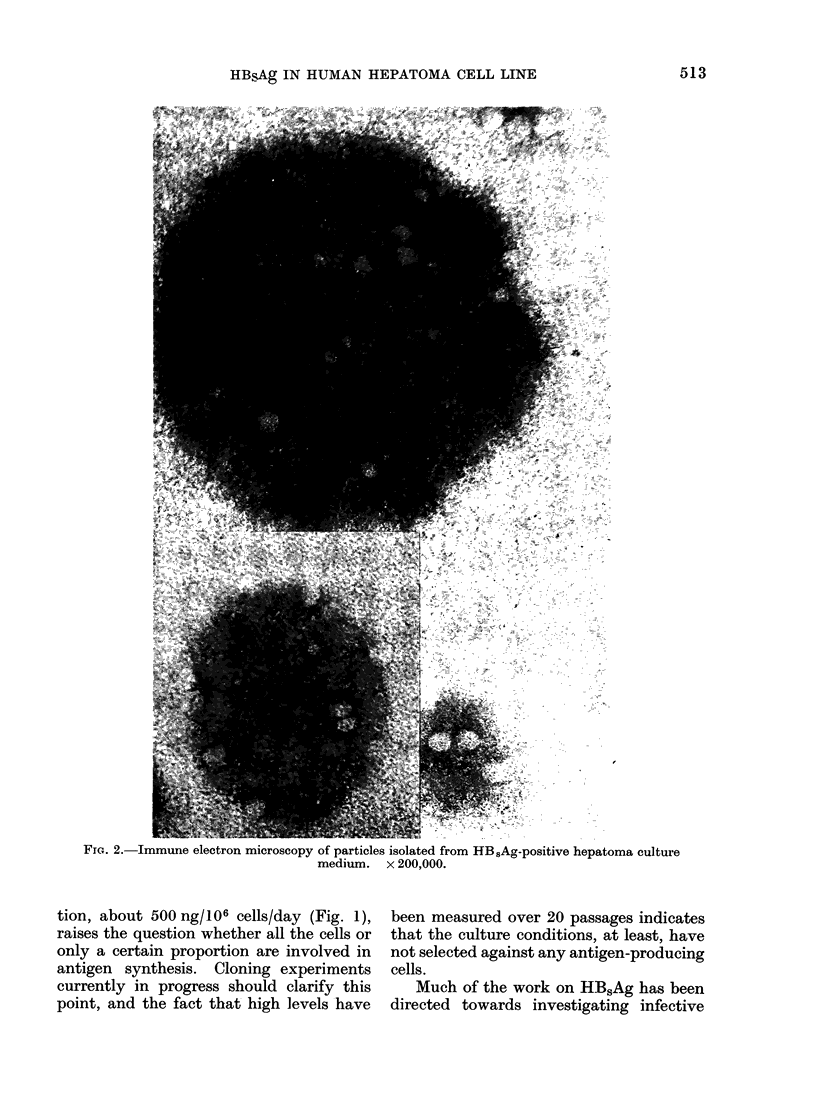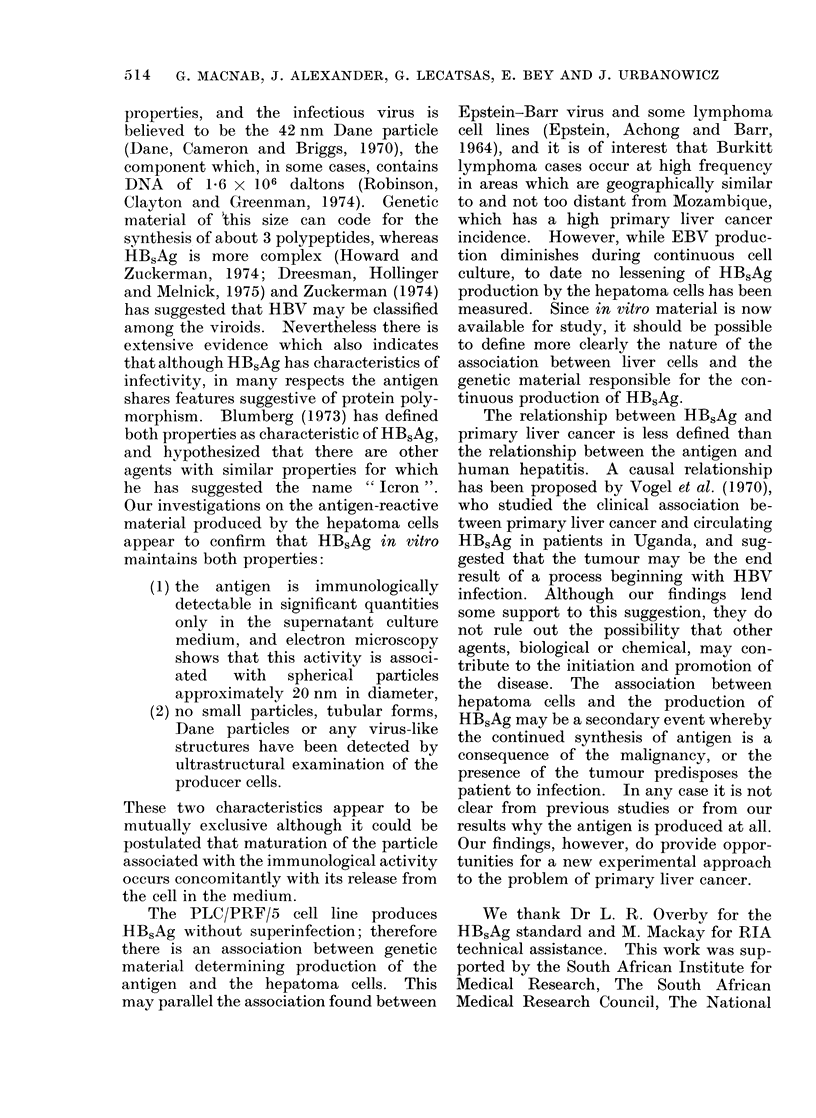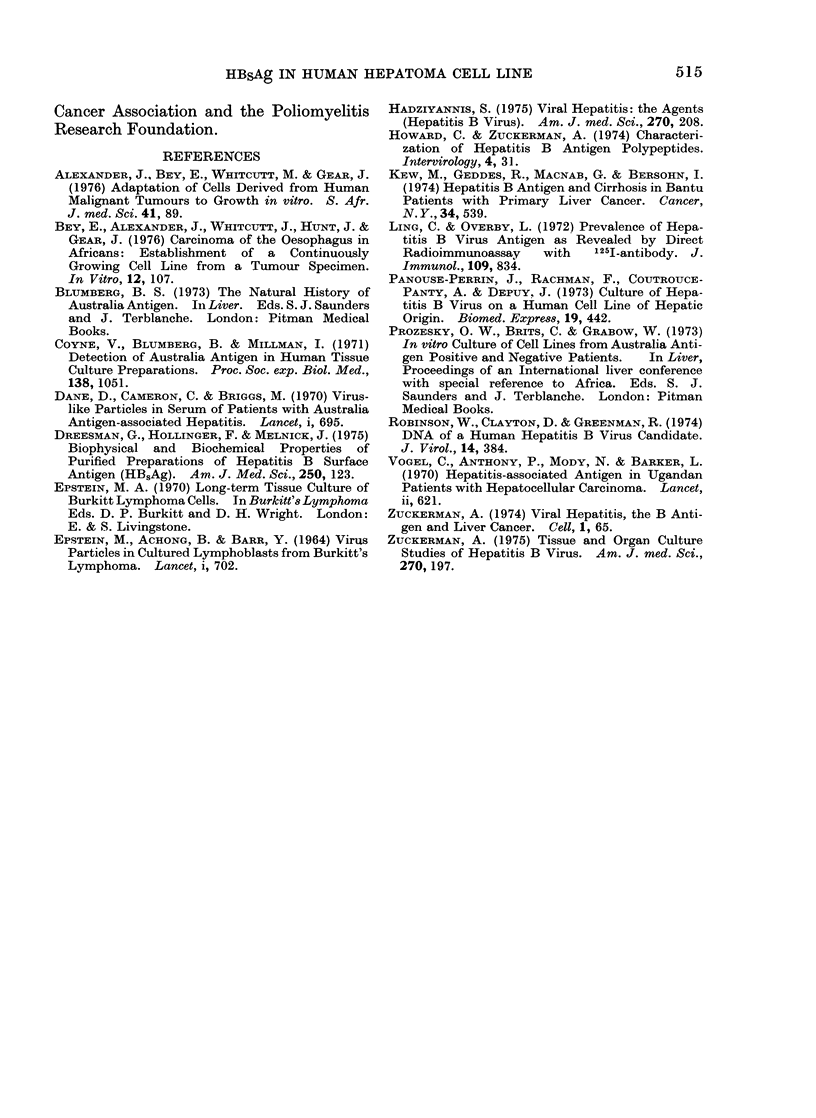# Hepatitis B surface antigen produced by a human hepatoma cell line.

**DOI:** 10.1038/bjc.1976.205

**Published:** 1976-11

**Authors:** G. M. MacNab, J. J. Alexander, G. Lecatsas, E. M. Bey, J. M. Urbanowicz

## Abstract

**Images:**


*Br. J. Cancer* (1976) **34**, 509

### HEPATITIS B SURFACE ANTIGEN PRODUCED BY A HUMAN HEPATOMA CELL LINE

G. M. MACNAB,* J. J. ALEXANDER,† G. LECATSAS,‡ E. M. BEY† AND J. M. URBANOWICZ*

*From the *Department of Serology, South African Institute for Medical Research, Johannesburg, †the Virus Cancer Research Unit, Poliomyelitis Research Foundation, Johannesburg, and ‡the Department of Microbiology, Institute of Pathology, University of Pretoria, Pretoria*

Received 31 March 1976 Accepted 1 July 1976

**Summary.—The human hepatoma cell line, PLC/PRF/5, was shown to produce hepatitis B surface antigen ($HB_SAg$). Immunologically reactive material was present in the supernatant tissue culture medium in significant amounts, and was associated with spherical particles approximately 20 nm in diameter. The rate of antigen production by the cells was estimated at 500 ng/day/$10^6$ cells by reference to a purified $HB_SAg$ standard. All immunological activity was neutralized by specific antibody and the subtype was ad. The studies reported here broaden the scope of investigations on both the *in vitro* production of $HB_SAg$ and the association between this antigen and primary liver cancer.**

THE CORRELATION between hepatitis B surface antigen ($HB_SAg$) and serum hepatitis has led to extensive research aimed at finding an *in vitro* system for the study of hepatitis B virus (HBV). A number of reports have appeared on the propagation of the virus in human liver cell and organ cultures (review by Zuckerman, 1975). After HBV infection, progressive changes in the cells have been noted and supernatant culture fluid could be passaged once or twice, but the short-term nature of the experiments and the lack of a constant source of tissue culture material has failed to provide, as yet, a standardized *in vitro* system for the study of this virus. Coyne, Blumberg and Millman (1971), using a different approach, cultured liver tissue from patients with $HB_SAg$ in their blood, and measured the antigen in the tissue culture fluid from 2 out of 23 separate culture preparations. Panouse-Perrin *et al.* (1973) inoculated a human hepatic cell line with $HB_SAg$-containing serum, and reported the replication of 25–27 nm particles, similar to the internal component of the Dane particle, through 14 passages in tissue culture. However, radioimmunoassays (RIA) for $HB_SAg$ were negative. Hadziyannis (1975) detected hepatitis B core antigen ($HB_CAg$) in liver slices taken from a patient with liver cell carcinoma and cirrhosis. The antigen was present in the cirrhotic tissue but not in the malignant cells. A human hepatoma cell line (Prozesky, Brits and Grabow, 1973) has been tested for the presence of $HB_SAg$, with negative results, and all attempts to inoculate the cultures with infectious material have shown no evidence of virus replication (Prozesky, personal communication).

The development of cell lines, either from normal human liver or hepatoma, still remains one possible means of supplying a constant source of material which will either support the replication of HBV or produce, *de novo*, antigens related to the virus. In view of the clinical association

Correspondence to: Dr G. M. Macnab, Department of Serology, South African Institute for Medical Research, P.O. Box 1038, Johannesburg, South Africa 2000.

between $HB_SAg$ and primary liver cancer (Vogel *et al.*, 1970; Kew *et al.*, 1974), and since the PLC/PRF/5 cell line was derived from a patient with primary liver cancer whose blood had circulating $HB_SAg$ (Alexander *et al.*, 1976) we tested this *in vitro* system for $HB_SAg$ reactivity. We have shown that this cell line produces $HB_SAg$ in significant amounts and that the antigen is similar to clinically derived material.

### MATERIALS AND METHODS

### (1) *Cell cultures and culture fluids*

All cells were cultured in 25-cm² flasks (Falcon Plastics). Growth medium (5 ml/flask) was Eagle's minimum essential medium containing 10% foetal bovine serum (Gibco) and 100 μg/ml each of penicillin and streptomycin. The PLC/PRF/5 hepatoma cells, which have been in continuous culture for 30 months, resemble hepatocytes and have a doubling time of 35 to 40 h *in vitro*. Ultrastructural examination has revealed no virus-like particles in these cells. As controls, both fresh growth medium and culture fluids harvested from other established human tumour cell lines were used. The control cell cultures included a carcinoma of the oesophagus (Bey *et al.*, 1976), a sarcoma (Alexander *et al.*, 1976) and two lymphomas, Raji and $EB_3$ (Epstein, 1970). Culture medium was harvested from the hepatoma and control cultures at various intervals. Hepatoma cells, mechanically scraped from the flasks, were disrupted by 4 cycles of freezing and thawing. All culture fluids and cell suspensions were clarified by centrifugation at 3000 *g* for 30 min prior to assay.

### (2) *$HB_SAg$ detection*

Solid phase RIA and passive reverse haemagglutination techniques were used (Abbott Laboratories, U.S.A., Ausria 11 and Hepnosticon; Burroughs Wellcome, Hepatest). RIA-negative results had count rates less than 0·3% of the radioactivity used. In order to compare results among samples assayed on different days the following procedure was employed:- positivity (P) was accorded to those samples having count rates per minute at least 5 standard deviations (s.d.) above the mean value (N) established for 7 negative controls (Ling and Overby, 1972). The cut-off value for the test series was 2·1 × N, and this factor is ample to allow for an increase of > 5 s.d. above the N value (Abbott Laboratories). Hence, positive results were computed from the formula P/N > 2·1. Known positive and negative sera were supplied by the manufacturers of the test kits. In addition, 5 sera from cases of proven viral hepatitis were included as positive controls.

*Quantitative analyses.*—For this purpose dilutions were made from an $HB_SAg$ standard of known concentration (1000 μg/ml, ad serotype, a gift from Dr Overby). Only dilutions containing between 7·8 and 250 ng/ml were used to generate a standard curve, because at greater concentration the relationship between $^{125}I$ ct/min and concentration was non-linear. The medium in 21 identical hepatoma cell cultures was renewed on Day 0, each receiving 5 ml of fresh medium. The culture fluids from 3 flasks were harvested separately every day for 6 days, and finally on the 8th day. The cells from each flask were suspended by 5-min incubation in 5 ml of 0·125% trypsin in phosphate-buffered saline containing 0·05% versene. The cells were pelleted, resuspended in 2 ml of medium, and counted in a haemocytometer. Each supernatant was assayed in triplicate, both undiluted and after ten-fold dilution.

### (3) *Preparation of culture medium for immune electron microscopy*

A neutralized ammonium sulphate solution was added, to give 50% saturation, to culture medium which had been in contact with the hepatoma cells for 6 days. The pellet obtained after centrifugation at 5000 *g* for 30 min was dissolved in distilled water to the original culture fluid volume and clarified by centrifugation at 12,000 *g* for 20 min. The supernatant was decanted and concentrated five-fold by filtration through an XM300 filter (Amicon). The concentrated material was layered on to an equal volume of a 55% saturated solution of CsCl and centrifuged at 45,000 rev/min in a SW50 head for 20 h.

Twenty-five-drop fractions were collected and dialysed against distilled water. Each gradient fraction was assayed for $HB_SAg$ reactivity (haemagglutination) and the fraction containing the highest activity was

TABLE.—*$HB_SAg$ Determination by Radioimmunoassay of Human Cell Line Supernatants, Hepatoma Cells and Positive Human Sera*

| Source of material | Age of supernatant in days | P/N values* Unconcentrated | 10× Concentrated |
|---|---|---|---|
| Hepatoma cells | — | 7·5 | 44·6 |
| Hepatoma cells supernatant | 3 | N.D. | 93·4 |
| Hepatoma cells supernatant | 3 | 26 | N.D.† |
| Hepatoma cells supernatant | 4 | 40 | N.D. |
| Hepatoma cells supernatant | 6 | 45·4 | N.D. |
| Hepatoma cells supernatant | 7 | 60·4 | N.D. |
| Oesophageal carcinoma supernatant | 4 | 1·5 | 1·4 |
| Lymphoma $EB_3$ supernatant | 3 | 1·2 | 1·1 |
| Lymphoma Raji supernatant | 3 | 1·1 | 1·1 |
| Sarcoma supernatant | 4 | 0·9 | 1·4 |
| Control medium | — | 1·0 | 1·5 |
| Positive serum 1 | | 55·6 | |
| 2 | | 17·6 | |
| 3 | | 4·9 | |
| 4 | | 9·9 | |
| 5 | | 52·0 | |

* $P/N = \frac{\text{ct/min experiment}}{\text{ct/min negative control}}$ P/N >2·1 taken as positive.

† Not done.

incubated for 16 h at 37°C with one fifth volume of rabbit antiserum to $HB_SAg$. The preparation was negatively stained with 3% phosphotungstic acid at pH6. Grids were viewed in a Philips EM 300 electron microscope.

### RESULTS

The table lists the values obtained for $HB_SAg$ reactivity in hepatoma cells and supernatants and from supernatants of other human cell lines. Results obtained from positive sera during the same period have been included for comparison. The assays were performed over a period of 4 weeks and, although the age of the harvested supernatants is listed, the number of cells in each culture was not evaluated and the material was harvested from different culture passages. All positive results (P/N > 2·1) were confirmed by passive reverse haemagglutination and specific neutralization. All the hepatoma supernatants tested were positive and the subtype was found to be ad in all cases. Since the disrupted cells had low activity compared with unconcentrated culture fluid, further assays were confined to the latter only.

As $HB_SAg$ activity appeared to increase with the age of the culture medium in contact with the hepatoma cells, experiments were designed to quantitate these levels, and the results of one of these tests is depicted in Fig. 1. The antigen in the supernatant does increase with time and the levels measured, by reference to the standard curve, indicate that approximately 500 ng of $HB_SAg$-reactive material is produced per day per $10^6$ cells.

Immune electron microscopy (Fig. 2) showed low-grade clumping of particles ranging in diameter from 16 to 24 nm, although the majority were approximately 20 nm in size. These particles are more or less spherical and there is some evidence of substructure. The CsCl gradient fraction which had the highest antigen activity and from which this material was prepared had a buoyant density of between 1·18 and 1·2 g/cm³. We have consistently found these structures in hepatoma culture supernatants. No Dane particles or tubular structures have been seen. All antigenic activity was precipitated from the supernatants with 50% ammonium sulphate saturation, and by centrifugation at 200,000 *g* for 4 h, but not at 100,000 for 2 h. All activity was retained by filtration through the XM300 filter.

FIG. 1.—*Radioimmunoassay of HB$_S$Ag in culture medium harvested from hepatoma cells.* 5 ml of supernatant harvested from each flask. (A) ○ average $^{125}$I ct/min/ml recorded from assay of 3 undiluted supernatant samples (1–8 days), ● number of cells/flask, average of 3 flasks. (B) ○ average $^{125}$I ct/min/ml for 3 supernatants assayed at 10× dilution (1–8 days), ▼ $^{125}$I ct/min from assay of serial dilutions of purified HB$_S$Ag of known concentration.

### DISCUSSION

The PLC/PRF/5 cell line consistently produces HB$_S$Ag at a significant level, and the antigen has the same characteristics as material derived from clinical sources. The table demonstrates that the levels of HB$_S$Ag measured in the supernatant culture medium are similar to those measured in serum from strongly antigenaemic patients and similar high readings have been obtained from supernatants tested over 9 months and 20 cell-culture passages. The consistent production of HB$_S$Ag of this cell line is in contrast to that in previous *in vitro* studies, where transient peak levels were detected following superinfection of liver cultures. Numerous cell lines, both established and semicontinuous, derived from liver and other tissues of both human and non-human origin, have been tested in attempts to establish an *in vitro* system for HBV research (Zuckerman, 1975). The results reported here demonstrate that the PLC/PRF/5 cell line provides a laboratory model for further studies on HB$_S$Ag production.

The estimated rate of HB$_S$Ag produc-

FIG. 2.—Immune electron microscopy of particles isolated from HB$_S$Ag-positive hepatoma culture medium. ×200,000.

tion, about 500 ng/$10^6$ cells/day (Fig. 1), raises the question whether all the cells or only a certain proportion are involved in antigen synthesis. Cloning experiments currently in progress should clarify this point, and the fact that high levels have been measured over 20 passages indicates that the culture conditions, at least, have not selected against any antigen-producing cells.

Much of the work on HB$_S$Ag has been directed towards investigating infective properties, and the infectious virus is believed to be the 42 nm Dane particle (Dane, Cameron and Briggs, 1970), the component which, in some cases, contains DNA of $1 \cdot 6 \times 10^6$ daltons (Robinson, Clayton and Greenman, 1974). Genetic material of this size can code for the synthesis of about 3 polypeptides, whereas $HB_SAg$ is more complex (Howard and Zuckerman, 1974; Dreesman, Hollinger and Melnick, 1975) and Zuckerman (1974) has suggested that HBV may be classified among the viroids. Nevertheless there is extensive evidence which also indicates that although $HB_SAg$ has characteristics of infectivity, in many respects the antigen shares features suggestive of protein polymorphism. Blumberg (1973) has defined both properties as characteristic of $HB_SAg$, and hypothesized that there are other agents with similar properties for which he has suggested the name " Icron ". Our investigations on the antigen-reactive material produced by the hepatoma cells appear to confirm that $HB_SAg$ *in vitro* maintains both properties:

(1) the antigen is immunologically detectable in significant quantities only in the supernatant culture medium, and electron microscopy shows that this activity is associated with spherical particles approximately 20 nm in diameter,
(2) no small particles, tubular forms, Dane particles or any virus-like structures have been detected by ultrastructural examination of the producer cells.

These two characteristics appear to be mutually exclusive although it could be postulated that maturation of the particle associated with the immunological activity occurs concomitantly with its release from the cell in the medium.

The PLC/PRF/5 cell line produces $HB_SAg$ without superinfection; therefore there is an association between genetic material determining production of the antigen and the hepatoma cells. This may parallel the association found between Epstein–Barr virus and some lymphoma cell lines (Epstein, Achong and Barr, 1964), and it is of interest that Burkitt lymphoma cases occur at high frequency in areas which are geographically similar to and not too distant from Mozambique, which has a high primary liver cancer incidence. However, while EBV production diminishes during continuous cell culture, to date no lessening of $HB_SAg$ production by the hepatoma cells has been measured. Since *in vitro* material is now available for study, it should be possible to define more clearly the nature of the association between liver cells and the genetic material responsible for the continuous production of $HB_SAg$.

The relationship between $HB_SAg$ and primary liver cancer is less defined than the relationship between the antigen and human hepatitis. A causal relationship has been proposed by Vogel *et al.* (1970), who studied the clinical association between primary liver cancer and circulating $HB_SAg$ in patients in Uganda, and suggested that the tumour may be the end result of a process beginning with HBV infection. Although our findings lend some support to this suggestion, they do not rule out the possibility that other agents, biological or chemical, may contribute to the initiation and promotion of the disease. The association between hepatoma cells and the production of $HB_SAg$ may be a secondary event whereby the continued synthesis of antigen is a consequence of the malignancy, or the presence of the tumour predisposes the patient to infection. In any case it is not clear from previous studies or from our results why the antigen is produced at all. Our findings, however, do provide opportunities for a new experimental approach to the problem of primary liver cancer.

We thank Dr L. R. Overby for the $HB_SAg$ standard and M. Mackay for RIA technical assistance. This work was supported by the South African Institute for Medical Research, The South African Medical Research Council, The National

Cancer Association and the Poliomyelitis Research Foundation.

REFERENCES

ALEXANDER, J., BEY, E., WHITCUTT, M. & GEAR, J. (1976) Adaptation of Cells Derived from Human Malignant Tumours to Growth *in vitro*. *S. Afr. J. med. Sci.* **41**, 89.

BEY, E., ALEXANDER, J., WHITCUTT, J., HUNT, J. & GEAR, J. (1976) Carcinoma of the Oesophagus in Africans: Establishment of a Continuously Growing Cell Line from a Tumour Specimen. *In Vitro*, **12**, 107.

BLUMBERG, B. S. (1973) The Natural History of Australia Antigen. In *Liver*. Eds. S. J. Saunders and J. Terblanche. London: Pitman Medical Books.

COYNE, V., BLUMBERG, B. & MILLMAN, I. (1971) Detection of Australia Antigen in Human Tissue Culture Preparations. *Proc. Soc. exp. Biol. Med.*, **138**, 1051.

DANE, D., CAMERON, C. & BRIGGS, M. (1970) Virus-like Particles in Serum of Patients with Australia Antigen-associated Hepatitis. *Lancet*, i, 695.

DREESMAN, G., HOLLINGER, F. & MELNICK, J. (1975) Biophysical and Biochemical Properties of Purified Preparations of Hepatitis B Surface Antigen ($HB_sAg$). *Am. J. Med. Sci.*, **250**, 123.

EPSTEIN, M. A. (1970) Long-term Tissue Culture of Burkitt Lymphoma Cells. In *Burkitt's Lymphoma* Eds. D. P. Burkitt and D. H. Wright. London: E. & S. Livingstone.

EPSTEIN, M., ACHONG, B. & BARR, Y. (1964) Virus Particles in Cultured Lymphoblasts from Burkitt's Lymphoma. *Lancet*, i, 702.

HADZIYANNIS, S. (1975) Viral Hepatitis: the Agents (Hepatitis B Virus). *Am. J. med. Sci.*, **270**, 208.

HOWARD, C. & ZUCKERMAN, A. (1974) Characterization of Hepatitis B Antigen Polypeptides. *Intervirology*, **4**, 31.

KEW, M., GEDDES, R., MACNAB, G. & BERSOHN, I. (1974) Hepatitis B Antigen and Cirrhosis in Bantu Patients with Primary Liver Cancer. *Cancer, N.Y.*, **34**, 539.

LING, C. & OVERBY, L. (1972) Prevalence of Hepatitis B Virus Antigen as Revealed by Direct Radioimmunoassay with $^{125}I$-antibody. *J. Immunol.*, **109**, 834.

PANOUSE-PERRIN, J., RACHMAN, F., COUTROUCE-PANTY, A. & DEPUY, J. (1973) Culture of Hepatitis B Virus on a Human Cell Line of Hepatic Origin. *Biomed. Express*, **19**, 442.

PROZESKY, O. W., BRITS, C. & GRABOW, W. (1973) *In vitro* Culture of Cell Lines from Australia Antigen Positive and Negative Patients. In *Liver*, Proceedings of an International liver conference with special reference to Africa. Eds. S. J. Saunders and J. Terblanche. London: Pitman Medical Books.

ROBINSON, W., CLAYTON, D. & GREENMAN, R. (1974) DNA of a Human Hepatitis B Virus Candidate. *J. Virol.*, **14**, 384.

VOGEL, C., ANTHONY, P., MODY, N. & BARKER, L. (1970) Hepatitis-associated Antigen in Ugandan Patients with Hepatocellular Carcinoma. *Lancet*, ii, 621.

ZUCKERMAN, A. (1974) Viral Hepatitis, the B Antigen and Liver Cancer. *Cell*, **1**, 65.

ZUCKERMAN, A. (1975) Tissue and Organ Culture Studies of Hepatitis B Virus. *Am. J. med. Sci.*, **270**, 197.